# The “Virtual Biopsy” of Cancerous Lesions in 3D: Non-Invasive Differentiation between Melanoma and Other Lesions Using Vibrational Optical Coherence Tomography

**DOI:** 10.3390/dermatopathology8040058

**Published:** 2021-12-13

**Authors:** Frederick H. Silver, Tanmay Deshmukh, Nikita Kelkar, Kelly Ritter, Nicole Ryan, Hari Nadiminti

**Affiliations:** 1 Department of Pathology and Laboratory Medicine, Robert Wood Johnson Medical School, Rutgers, The State University of New Jersey, Piscataway, NJ 08854, USA; 2 OptoVibronex, LLC., Allentown, PA 18104, USA; tmd24895@gmail.com (T.D.); nuk1@scarletmail.rutgers.edu (N.K.); 3 Dermatology, Summit Health, Berkeley Heights, NJ 07922, USA; KRitter@summithealth.com (K.R.); nryan@summithealth.com (N.R.); HNadiminti@summithealth.com (H.N.)

**Keywords:** collagen, fibrous tissue, stroma, actinic keratosis, basal cell carcinoma, squamous cell carcinoma, melanoma, epithelial-mesenchyme transition, extracellular matrix

## Abstract

Early detection of skin cancer is of critical importance to provide five year survival rates that approach 99%. By 2050, one out of five Americans by age 70 will develop some form of skin cancer. This will result in a projected rate of 50 million skin biopsies per year given the current rate of escalation. In addition, the ability to differentiate between pigmented lesions and melanomas has proven a diagnostic challenge. While dermoscopy and visual analysis are useful in identifying many skin lesions, additional non-invasive techniques are needed to assist in the analysis of difficult to diagnose skin tumors. To augment dermoscopy data, we have developed 3D maps based on physical biomarker characteristics of benign and cancerous lesions using vibrational optical coherence tomography (VOCT). 3D images based on quantitative physical data involving changes in cellular and fibrous tissue stiffness along with changes in vascular quality are used to map and evaluate different types of cancers. 3D tumor maps constructed using quantitative VOCT data and OCT images have been used to characterize the differences between melanoma and other lesions. These characteristics can be used to plan the excision of difficult lesions where extensive surgery may be needed to remove the entire tumor in one step. In addition, it is now possible to use dermoscopy and VOCT to non-invasively differentiate between different cancerous lesion types using measurements of the resonant frequency of new cellular and vascular peaks. Quantitative VOCT information along with dermoscopic findings can be collected and analyzed remotely using artificial intelligence to improve cancerous tissue diagnosis.

## 1. Introduction

Approximately 5 million patients develop skin cancer in the United States each year [1,2,3] and current predictions are that by age 70, one out of five Americans will develop some form of skin cancer resulting in a projected 50 million skin biopsies performed per year given the current rate of escalation [1,2,3,4]. Skin lesion analysis is performed by dermatologists through visual or dermoscopic evaluation [5,6,7]. The use of dermoscopy in telemedicine if augmented using other non-invasive techniques would improve non-invasive lesion diagnosis. However, current techniques have a poor diagnostic ability for melanoma and millions of benign skin biopsies (55% of the total) are performed each year [8,9,10]. Therefore, because of the large volume and cost for performing biopsies on benign lesions, it is important to evaluate alternative methods for deciding which tissues to biopsy and perform histopathology on. In addition, once physical parameter “fingerprints” of different cancerous lesions are developed, this will provide additional information that will assist the physician to distinguish between pigmented lesions and melanomas.

### 1.1. Diagnostic Criteria for Different Skin Pre- and Cancerous Lesions

#### 1.1.1. Basal Cell Carcinoma

Basal cell carcinoma (BCC) is the most prevalent form of skin cancer worldwide. It accounts for 90% of all skin cancers in the United States [1,11]. The predominant form is nodular BCC that accounts for 50% of all of these lesions; it is characterized by aggregates of basaloid cells with well-defined borders. These well-defined borders appear to contain extracellular matrix (ECM) with prominent vessels [11,12,13] and collagen [14]. Vibrational optical coherence tomography (VOCT) study results indicate that a fibrotic peak that characterizes BCC lesions is not present in normal skin [15] and another vascular peak appears to be present that may reflect the presence of new blood vessels [12,13]. Superficial BCC is another common variant of BCC. It is characterized by nests of basaloid cells that extend from the epidermis, with neoplastic cells that resemble primordial germ cells. Peripheral palisading is usually prominent and tumor islands show a well demarcated border [16].

#### 1.1.2. Actinic Keratosis

Actinic keratosis (AK) is considered an early form of SCC [1,2]. Pathologically, AK is characterized by a horizontal alteration of parakeratotic and orthokeratotic hyperkeratosis, with an atrophic or acanthotic epidermis [13]. Neoplastic keratinocytes in the basal layer show increased cellularity, nuclear pleomorphism, and scattered mitoses [13].

#### 1.1.3. Squamous Cell Carcinoma

Conventional squamous cell carcinoma (SCC) is characterized by atypical cells in the dermis, showing enlarged and pleomorphic nuclei with atypical mitotic activity [13]. Inflammation is usually present in ulcerated lesions and typically consists of lymphocytes, plasma cells, and neutrophils. Keratinous pearls are present on the dermis, surrounded by nests of atypical cells and reduced stroma with lymphocytes [13,17].

#### 1.1.4. Melanoma

Small melanocytic lesions remain a significant diagnostic challenge not only for clinicians but also for pathologists. Progressive smaller lesions are excised under the screening of dermoscopic features [18,19]. According to Carli and colleagues, dermoscopy is not a useful tool for the diagnosis of lesions up to 6 mm in diameter compared to its use for screening larger lesions [19]. This observation is in agreement with another study, in which only 1 out of 76 suspicious lesions was diagnosed as malignant [19]. Ordinary benign melanocytic lesions usually are not clinically suspicious. Clearly, the diagnosis of melanoma is a difficult challenge and any new technique that provides additional criteria to help make the diagnosis is warranted.

The purpose of this paper is to present virtual biopsies of AK, BCC, SCC and melanoma. The virtual biopsy when augmented by physical VOCT data on the stiffness of cells, dermal collagen, fibrotic collagen, and new vascular tissue can be used to construct 3D tumor images to differentiate benign from cancerous lesions. This quantitative data along with dermoscopy can be used to diagnose lesions via telemedicine, as well in artificial intelligence based analysis of cancerous lesions.

VOCT is a new technique that combines optical coherence tomography (OCT) imaging with measurements of cellular, collagen, and vascular elastic moduli. This is achieved by non-invasively applying infrared light and audible sound transversely to the tissue and measuring the resonant frequency of the resultant vibrational waves based on the reflected light from the surface. Using this technique, both camera images, as well as OCT images, are obtained. In addition, quantitative physical biomarkers of cellular, collagen, vascular and fibrotic tissue are measured. Recent study results indicate that cancerous skin lesions present new vibrations at 80 Hz, 130 Hz and 260 Hz representing changes in cellular, vascular and fibrotic peaks [17,20,21,22,23,24,25,26].

## 2. Materials and Methods

### 2.1. Subjects

**Figure 1:**
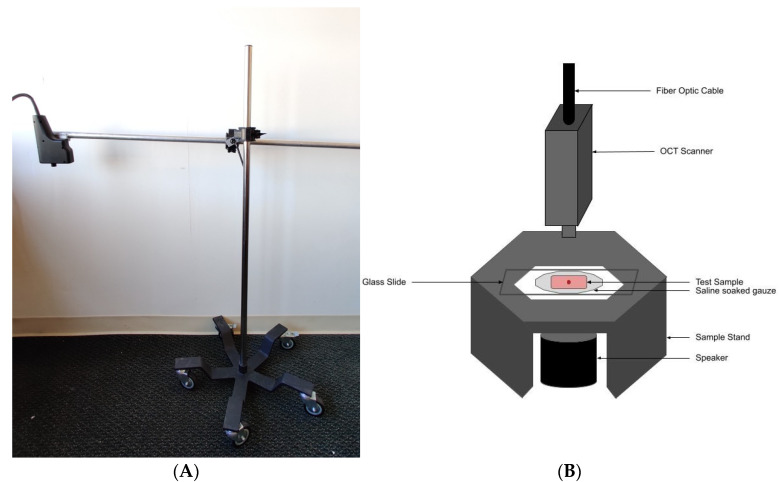
Picture of universal hand piece stand (**A**) used in conjunction with a table mounted speaker stand for making OCT and VOCT measurements on control skin in vivo. (**B**) Diagram of microscope stand used to mount hand piece, sample support, holder and speaker used to make OCT and vibrational OCT (VOCT) measurements on skin lesion biopsies. The blue tooth speaker is activated by an app that creates a sinusoidal signal generated by an I5 computer contained in the OCT device. The VOCT measurement of resonant frequency is made by processing the raw vibrational amplitudes as a function of time created by the sinusoidal driving signal after all out-of-phase data is filtered out. The filtering results in only elastic amplitudes being used to calculate the resonant frequency and modulus of the sample studied.

Normal skin from 14 subjects (11 males and three females) was studied in vivo using VOCT after informed consent was obtained. Control skin examined included skin from the hands, arms and legs. The resonant frequencies of the components of skin were measured in vivo by mounting the OCT hand piece on a custom built universal mount that was supported over the area of skin to be studied (Figure 1A). The subjects studied ranged in age from 21 to 71 years old and both camera and OCT images of the lesion were recorded.

Suspicious skin lesions identified by dermoscopy in the Dermatology Clinic at Summit Health (Berkeley Heights, NJ, USA) were biopsied and studied in vitro using VOCT on a custom built sample stand (Figure 1B). These lesions appeared during dermoscopy to contain layers of cells, superficial blood vessels, or abnormal color. They were obtained from the arms, legs, abdomen and necks of subjects at Summit Health. The biopsied lesions were studied blindly by VOCT without identification of the age and sex of the patient. Forty biopsies of complete excisions and fifty one Mohs sections were examined using VOCT as part of an IRB approved study. Several of the biopsies were large enough to make multiple measurements on different areas of each sample. VOCT measurements were made on an area of about 0.0625 mm^2^. All subjects signed consent forms prior to enrolling in the IRB approved study.

### 2.2. OCT Images and Scans of Pixel Intensity Versus Depth

OCT image collection was accomplished using a low cost Lumedica Spectral Domain OQ 2.0 Labscope (Lumedica Inc., Durham, NC, USA) operating in the scanning mode at a wavelength of 840 nm. The device generates a 512 × 512-pixel image with a transverse resolution of 15 micrometers and an A-scan rate of 13,000/s. All gray scale OCT images were color coded to enhance the image details. For the pixel intensity versus depth plots, the surface of the sample was traced and the averages of pixel values were calculated along the surface of the sample, which were then plotted against the depth. For curved biopsy specimen, the tracing was done parallel to the surface of the image.

Multiple cross-sectional OCT scans were collected using the volume scan software included in Lumedica OQ Labscope and processed into 3D images using image J software. All lesions were photographed using the camera mounted in the instrument handpiece.

The pixel intensities obtained from the gray scale images were plotted versus depth for each sample studied. The enhanced OCT images used darker colored (blue and purple) regions to reflect lower pixel intensities while the lighter (yellowish) regions reflected higher pixel intensity regions. Pixel intensities were processed using image J software, analyzed with a MATLAB program, and plotted versus skin depth. Previous studies have shown that the images of normal skin and cancerous lesions seen by OCT correlate with the histological images seen in sections cut from tissue biopsies [17,20,21].

### 2.3. Measurement of Resonant Frequency and the Elastic Modulus

The OQ Labscope was modified as shown in Figure 1B by adding a 2 inch diameter speaker to vibrate the sample in the VOCT studies. The Labscope was also modified to collect and store single raw image data that was used to calculate sample displacements (amplitude information) from A line data. The data were processed using MATLAB software as discussed previously [17,20,21,22,23,24,25,26]. The displacement of the tissue is detected by measuring the frequency dependence of the deformation based on the reflected infrared light and filtered to collect only vibrations that were in phase (elastic component) with the input sound signal. The vibrations for each frequency are isolated to calculate the amplitude. These amplitudes are plotted against the frequency of the vibrations. The result is a spectrum of displacements for specific tissue components as a function of the frequency of the applied sound; the resonant frequency of each tissue component has been assigned previously based on studies on a variety of soft tissues and polymeric materials [17,20,21,22,23,24,25,26].

The resonant frequency of a tissue component is defined as the frequency at which the maximum in-phase displacement is observed in the amplitude data. The measured resonant frequencies are converted into elastic modulus values using a calibration equation, Equation (1) developed based on in vitro uniaxial mechanical tensile testing and VOCT measurements made on the same tissue as reported previously [20,21,22,23,24,25,26]. The resonant frequency of each sample is determined by measuring the displacement of the tissue resulting from sinusoidal audible sound driving frequencies ranging from 30 Hz to 300 Hz, in steps of 10 Hz. The peak frequency (the resonant frequency), *f_n_*, is defined as the frequency at which the displacement is maximized.

(1) $$\mathrm{Soft}\;\mathrm{Tissues}\;E\times d=0.0651\times \left(fn^{2}\right)+233.16$$

Calibration studies using in vitro uniaxial tensile testing and VOCT measurements are used to develop Equation (1) for soft tissues. Since most soft tissues have a density very close to 1.0, Equation (1) is valid for the majority of tissues found in the body; where the thickness d is in m and is determined from OCT images, $fn^{2}$ is the square of the resonant frequency, and E is the elastic modulus in MPa as discussed previously [20,21,22,23,24,25,26].

Normal skin studies were conducted in vivo using the universal hand piece mount shown in Figure 1A. Tissue biopsies were studied by VOCT in vitro using the microscope stage shown in Figure 1B within 5 min of harvesting by the Dermatologist and kept wet using moist saline impregnated gauze during testing.

Once VOCT studies were conducted, the biopsy samples were immersed in fixative and transported to the pathology lab for diagnosis. Histopathology on skin biopsies was conducted by a dermatopathologist after routine dehydration in alcoholic solutions, embedding in paraffin, thin sectioning and staining with H&E. Mohs thin sections were processed after fixation by frozen sectioning and H&E staining. They were reviewed by a trained Mohs dermatopathologist who conducted the pathological analysis.

## 3. Results

### 3.1. Resonant Frequency Peaks of Normal Skin

**Figure 2:**
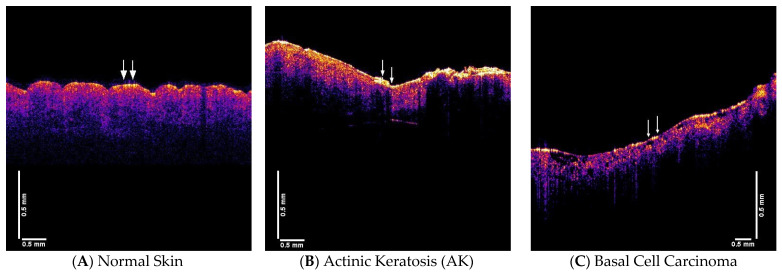

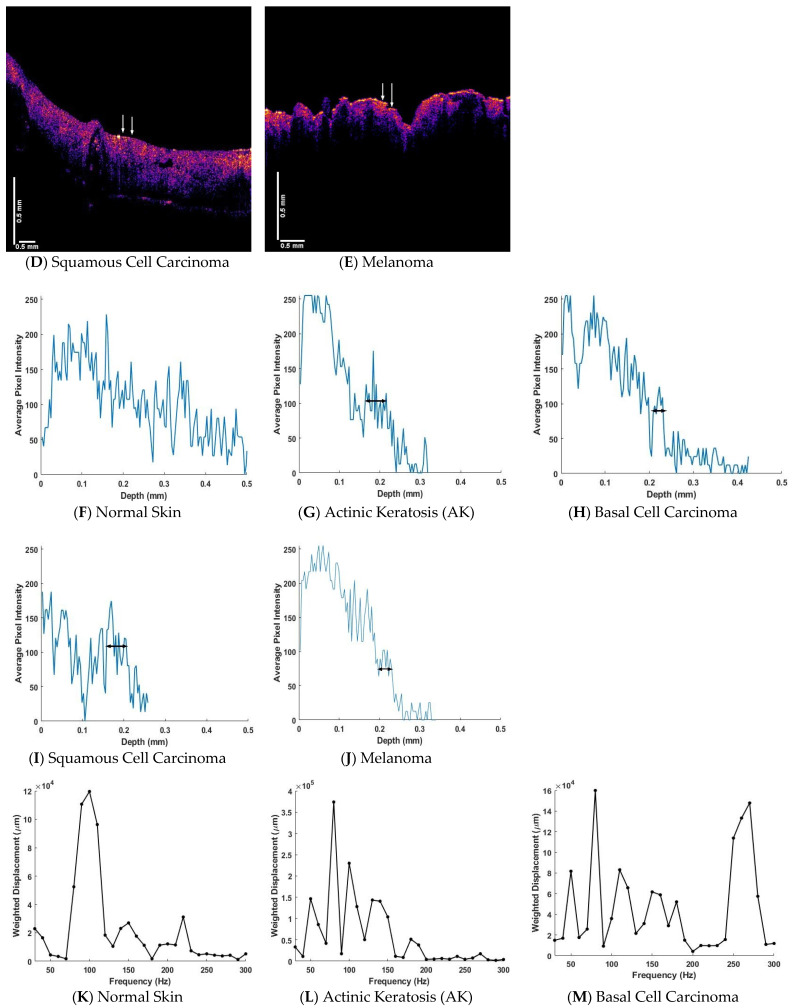

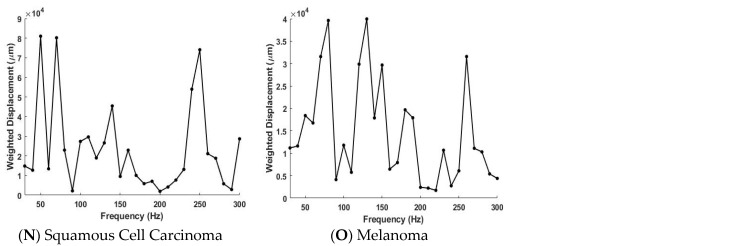
This figure shows a compilation of typical OCT images, pixel intensity versus depth plots and weighted displacement versus frequency plots for normal skin, AK, BCC, SCC and melanoma. The color coded OCT images are representative images: (**A**) normal skin, (**B**) AK, (**C**) BCC, (**D**) SCC, and (**E**) melanoma of the lesions observed in our studies. The pixel intensity versus depth of: (**F**) normal skin, (**G**), AK (**H**), BCC, (**I**) SCC, and (**J**) melanoma are shown below the color coded images and the arrows show where the lesion was studied by VOCT. Plots of weighted displacement versus frequency of: (**K**) normal skin, (**L**) AK, (**M**) BCC, (**N**) SCC, and (**O**) melanoma are shown below the pixel intensity data. This data was collected on the tissues shown in A through E at locations between the arrows. The weighted displacement is normalized by dividing the experimentally observed displacement of the sample by the displacement of the speaker in the absence of the sample. The horizontal lines shown in G through J in Figure 2 correspond with the location of the black reflective spots in the OCT images (Figure 2B–E). While AK (Figure 2L) does not have a significant resonant frequency peak at 260 Hz it does have one at about 180 Hz. BCC, SCC and melanoma all have large resonant frequency peaks at about 260 Hz that coincide with the location of the black spots in the OCT images C through E. In AK, the peak at 180 Hz may represent the beginning of fibrous tissue deposition with a stiffness of about 5 MPa, while the peak at 260 Hz in BCC, SCC and melanoma may represent mature fibrous tissue with a stiffness of about 15 MPa. Note the smaller resonant frequency peaks in BCC, SCC and melanoma between 150 and 180 Hz appear similar to the 180 Hz peak in AK.

Normal skin is characterized by major resonant frequency peaks at 50 Hz (dermal cells), and 100 Hz (dermal collagen) as shown in Table 1 and Figure 2 [15]. In contrast, AK lesions have additional peaks at 80 Hz and 130 Hz; while BCC, SCC and melanoma have resonant frequency peaks at, 80 Hz, 130 Hz and 260 Hz (Table 1). In this study, we used VOCT measurements to characterize individual lesion types and compare the results to melanomas. In addition, reconstructed 3D images of different lesions using pixel intensity versus depth plots, measured values of the elastic modulus as well as volume scans showing pixel intensity versus depth plots are presented for each type of lesion (Figure 2).

### 3.2. OCT Images, Pixel Intensity Vesus Depth and Weighted Displacement vesus Frequency Data

Figure 2 shows a compilation of typical OCT images, pixel intensity vesus depth plots and weighted displacement versus frequency plots for normal skin, AK, BCC, SCC and melanoma. The color coded OCT images are representative images: (A) normal skin, (B) AK, (C) BCC, (D) SCC, and (E) melanoma of the lesions observed in our studies. The pixel intensity vesus depth of (F) normal skin, (G), AK (H), BCC, (I) SCC, and (J) melanoma are shown below the color coded images and the arrows show where the lesion was studied by VOCT. Plots of weighted displacement versus frequency of (K) normal skin, (L) AK, (M) BCC, (N) SCC, and (O) melanoma are shown below the pixel intensity data. This data was collected on the tissues shown in A through E at locations between the arrows.

The weighted displacement is normalized by dividing the experimentally observed displacement of the sample by the displacement of the speaker in the absence of the sample. The horizontal lines shown in G through J in Figure 2 correspond with the location of the black reflective spots in the OCT images (Figure 2B–E). While AK (Figure 2L) does not have a resonant frequency peak at 260 Hz it does have one at about 180 Hz. BCC, SCC and melanoma (Figure 2M–O) all have large resonant frequency peaks at about 260 Hz that coincide with the location of the black spots in the OCT images C through E. In AK, the peak at 180 Hz may represent the beginning of fibrous tissue deposition with a stiffness of about 5 MPa, while the peak at 260 Hz in BCC, SCC and melanoma may represent mature fibrous tissue with a stiffness greater than 15 MPa. Note the smaller resonant frequency peaks in BCC, SCC and melanoma at 180 Hz appear similar to the 180 Hz peak in AK.

The plots of weighted displacement versus frequency are very similar for BCC, SCC and melanoma (see Figure 2M–O). The plot for normal skin has prominent resonant frequency peaks at 50 Hz, 100 Hz and 150 Hz. Each of the plots for cancerous lesions show resonant frequency peaks at 80 Hz, 130 Hz, and 260 Hz that are not present in normal skin (Table 1).

### 3.3. Virtual Biopsy Reconstructions of Normal Skin, AK, BCC, SCC and Melanoma

**Figure 3:**
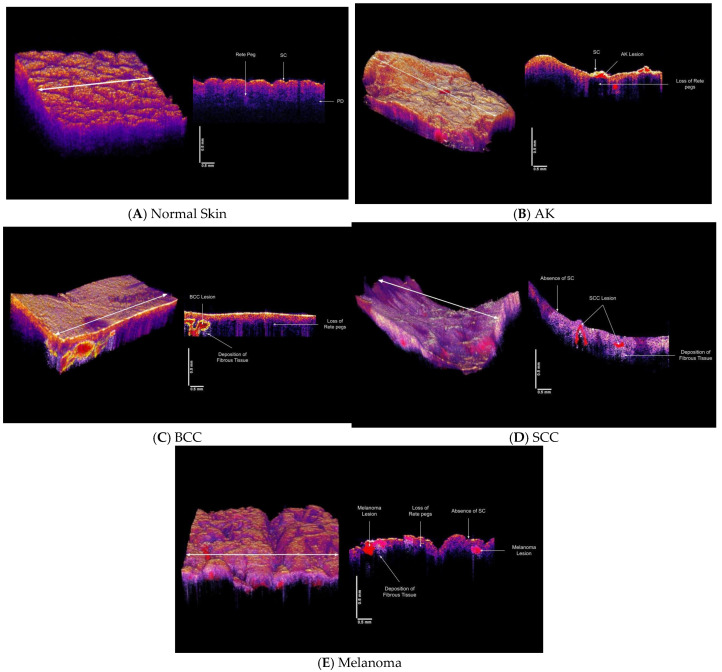
3D reconstructions of normal skin (**A**), AK (**B**), BCC (**C**), SCC (**D**) and melanoma (**E**) made using OCT images, pixel intensity versus depth data and weighted displacement versus frequency data shown in Figure 2. 3D normal skin reconstruction (**A**) in vivo on the left was created using the volume scan app on the Lumedica OCT and a cross-section of the skin cut at the arrow on the 3D reconstruction in (**A**) (right). The location of the stratum corneum (SC) and rete pegs and the papillary dermis (PD) were identified from the OCT image as reported previously [17,20,21]. An AK lesion (**B**) 3D reconstruction (left) with a cross-section of the lesion on the right. Note the absence of the rete pegs in some areas of the lesion and the presence of the lesion in red. The location and characterization of the lesion were based on the pixel intensity vesus depth plot (Figure 2G) and the weighted displacement versus frequency plot shown in Figure 2L. There is a fibrosis peak at about 180 Hz which is similar to that seen in healing wounds. (**C**) 3D reconstruction of a biopsy with a BCC lesion shown in red (left). The location of the lesion was identified based on data of Figure 2H and the composition was evaluated based on data of Figure 2M. Note the loss of the rete pegs in the cross-section on the left as well as the presence of nodular lesion at the epidermal-dermal junction. Fibrous tissue is deposited below the BCC lesion. (**D**) 3D reconstruction of an SCC (left) based on data shown in Figure 2I,N showing loss of stratum corneum and the presence of SCC lesions (right) and fibrous tissue below the lesion. Note the absence of the rete pegs in some areas and changes in the epidermal-dermal junction. (**E**) 3D reconstruction of a melanoma (right) showing lesions in red on right, and loss of stratum corneum, rete pegs and deposition of fibrous tissue below lesion involving both epidermis and dermis. The melanoma lesion was reconstructed using data from data in Figure 2J,O.

3D virtual biopsy reconstructions of normal skin, AK, BCC, SCC and melanoma are shown in Figure 3. These maps were made using the locations of the fibrous tissue from the pixel intensity versus depth plots. It was assumed that the black spots in the OCT images corresponded with the plateau in the pixel intensity versus depth plots and are the location of the newly deposited fibrous tissue with resonant frequencies between about 180 and 260 Hz. This depth is the approximate location of the epidermal-dermal junction in the skin. Based on the location of the plateaus in Figure 2L–O and the 260 Hz peak in the weighted displacement versus frequency plot, the fibrous tissue occurs at 0.2 to 0.25 mm in BCC, at 0.15 to 0.25 mm in SCC, and at 0.2 to 0.25 in melanoma all close to the border between the epithelial and dermal interfaces. Due to the limitations of the resolution of the OCT used in this study, the location for the blood vessels for both new (130 Hz) and old (150 Hz) components could not be mapped in this study.

Table 1 compares the resonant frequencies and moduli for cellular, new vascular and fibrous collagen resonant frequencies and moduli for normal skin, AK, BCC, SCC and melanoma. While normal skin has major resonant frequency peaks at 50 Hz (cellular) AK has an additional peak at 80 Hz. BCC, SCC and melanoma have peaks at 80, 130 and 260 Hz that are not present in normal skin. In addition, the data in Table 2 illustrates that using the ratio of the 80 to 130 resonant frequency peak heights, melanoma can be differentiated from BCC and SCC with a *p*-value of about 0.02.

## 4. Discussion

The use of visual examination and dermoscopic evaluation has enhanced the dermatologist’s ability to detect suspicious lesions. The addition of VOCT, which can be done remotely with the aid of a trained technician, is a means of collecting additional quantitative lesion data that can be used along with dermoscopy in making a diagnosis of difficult lesions. The goal in improving skin cancer detection is to be able to differentiate between benign lesions and cancers, specifically melanoma, prior to extensive skin removal. While most cancers are diagnosed histologically based on the presence of mitotic figures and abnormal cellular nuclear morphology, it is now becoming apparent from the literature that changes in the ECM around skin lesions occur in pre-cancerous and cancerous lesions [17,27,28]. These changes are quantitative physical biomarkers of cancer and may be useful in the early detection of cancerous lesions as well as in using artificial intelligence in assisting in lesion diagnosis.

Histopathology shows significantly higher microvessel densities in the peritumor stroma of BCCs when compared to normal skin or benign tumors. The quantification of peritumor microvessels may further assist with tumor evaluation [11]. Angiogenesis is required for a tumor to grow beyond a size of ~1–2 mm and to metastasize [29,30,31]. While normal vessels are tethered to surrounding extrcellular matrix (ECM) [32], new tumor blood vessels are more friable and appear to have lower moduli than those of normal blood vessels. While these new vessels can be imaged by high-resolution OCT, it is now possible with VOCT to provide evidence of their existence based on the new vibrational peak at 130 Hz.

Beyond the appearance of new blood vessels, it has been shown that the angiogenic phenotype creates a boundary line between hyperplasia and neoplasia [29,30,31]. Even though vascular patterns vary significantly in solid tumors, there is a certain relationship between tumor growth and the degree of vascularization [33]. While a number of different vascular morphologies have been identified by dermoscopy, OCT, confocal microscopy and histopathology [11], the relationship between the altered vascular morphology and fibrotic deposition surrounding skin cancers is still unknown. However, the appearance of a new cellular peak at 80 Hz and the deposition of fibrotic tissue around tumors increases the stiffness of the ECM and is the parameter that can be used to provide a physical biomarker to identify skin cancers [34]. Beyond this increase in cellular stiffness associated with lesion formation is what appears to be a continuum of deposition of fibrous tissue with a modulus of about 5 MPa (AK, pre-cancerous lesion) that begins as a result of wound healing and an epithelial-mesenchymal transition, and ends up in the deposition of mature stiff fibrous tissues with a modulus of greater than 15 MPa (BCC, SCC and melanoma, cancerous lesions).

While OCT alone does not have enough resolution to identify mitotic cells and abnormal nuclear material, VOCT can assess the “quality” of the vasculature and newly deposited collagen around the tumor. The cancer associated vasculature appears to be less stiff than the vessels normally present in the skin perhaps due to the absence of tethering of these new friable vessels to the surrounding veins and nerves. This tethering may be absent in skin cancerous lesions. The deposition of fibrous tissue surrounding skin cancerous tissue may suggest that the stiff fibrous tissue may be a pathway for cellular migration and metastasis especially if the new fibrous tissue replaces the basement membrane that normally separates the epidermis and dermis. It is interesting to note that the stiffness of fibrous tissue in normal scars and AK is about 5 MPa, while that for BCC, SCC and melanoma jumps up to about 15 MPa (see Table 1). This large increase in cell stiffness and fibrotic tissue in cancerous tissue explains the published observations that cancerous cells and tissue are stiffer than normal tissues [35,36,37,38].

Using VOCT technology, 3D tumor maps can be constructed based on the stiffness of the component cells and tissues and virtually serially sectioned (Figure 3) prior to excision of biopsies or samples being sent out for diagnosis by a pathologist. These maps along with dermoscopic observations can be used to identify melanomas as well as to define the margins of a lesion remotely. Maps can be constructed from measurements done directly on the patient prior to lesion excision in cases where extensive surgery may be needed to remove the entire tumor in one step. VOCT and dermoscopic observations in conjunction with artificial intelligence can be used to diagnose difficult skin lesions and improve the clinical utility of teledermoscopy [39].

## 5. Conclusions

The location and physical biomarker characteristics of skin cancers are different than those found in normal skin and benign lesions and can be identified virtually using VOCT. While benign skin lesions have major peaks at 50 and 100 Hz, pre-cancerous lesions have additional peaks at 80 and 130 Hz, and cancerous lesions have additional peaks at 80, 130 and 260 Hz. 3D images created based on changes in cellular and fibrous tissue stiffness along with changes in vascular quality can be used to map and evaluate different types of cancers including melanoma. While VOCT cannot identify changes at the cellular level, it can quantitatively identify the new vessel and fibrous tissue deposition that are associated with cancerous tumor growth. Based on the results of this pilot study it can used to differentiate between melanomas and other cancerous lesions.

3D tumor maps constructed from VOCT data and the location of the height of the 80, 130 and 260 Hz peaks and OCT images can be created prior to surgery from non-invasive measurements. They can be made directly on the patient and be used to plan the removal of difficult skin lesions where extensive surgery may be needed to remove the entire tumor in one step.

## Figures and Tables

**Table 1:** Mean resonant frequency peaks in Hz and moduli in MPa for normal skin, AK, BCC, SCC and melanoma based on VOCT data. The standard deviations are shown in parentheses. Note multiple measurements of resonant frequency and modulus were made on some of the large biopsies.

	Normal Skin	AK	BCC	SCC	Melanoma
New Resonant Frequency Peak in Hz for Different Skin Lesions					
Sample size	14	7	55	46	57
50 Hz	50 {0}	50 {0}	49.09 {2.90}	49.56 {2.06}	50 {0}
80 Hz	NA	75.71 {7.68}	76.72 {4.733}	75.86 {4.97}	76.31 {4.86}
130 Hz	NA	127.14 {4.81}	126 {4.94}	127.82 {4.17}	128.24 {3.83}
260 Hz	NA	NA	262.90 {4.58}	262.82 {4.55}	262.98 {4.61}
Modulus Data in MPa for Different Components in Skin Lesions					
Sample size	14	7	55	46	57
50 Hz	0.93 {0.057}	1.12 {0.25}	1.02 {0.096}	1.02 {0.075}	1.46 {0.162}
80 Hz	NA	1.75 {0.23}	1.80 {0.27}	1.74 {0.21}	2.26 {0.29}
130 Hz	NA	4.52 {1.12}	4.05 {0.605}	4.02 {0.39}	4.81 {0.56}
260 Hz	NA	NA	15.95 {2.40}	15.46 {1.75}	17.44 {1.94}

**Table 2:** Comparison of p values for the ratio of the 130/80 Hz resonant frequency peaks when comparing different skin cancers for BCC, SCC and melanoma. Sample size reflects multiple measurements made on several locations on biopsies containing large lesions. All lesions were identified by black spots in the OCT images (see Figure 2A–E) and VOCT measurements were made on those areas marked by arrows in Figure 2A–E and identified from data shown in Figure 2K–O.

130/80 Hz Peak Ratios			
	Melanoma	BCC	SCC
Sample Size	57	55	46
Average {SD}	0.87 {0.58}	1.38 {1.26}	1.68 {1.61}
Melanoma	NA	**0.022**	**0.0043**

Values in bold represent statistically significant *p*-values.

## Data Availability

The data supporting the results can be found at optovibronex.com.
